# P-217. Neurocognitive Effects of Transitioning to Dolutegravir-based Antiretroviral Therapy in People with HIV in sub-Saharan Africa

**DOI:** 10.1093/ofid/ofaf695.439

**Published:** 2026-01-11

**Authors:** Henry Michael, Hannah Kibuuka, John Owuoth, Valentine Sing’oei, Jonah K Maswai, Reginald R Gervas, Abdulwasiu Tiamiyu, Zahra Parker, Trevor A Crowell, Victor Valcour, Julie A Ake, Frasia Oosthuizen

**Affiliations:** University of KwaZulu-Natal, Durban, KwaZulu-Natal, South Africa; Makerere University Walter Reed Project, Kampala, Kampala, Uganda; Henry M. Jackson Medical Research International, Kisumu, Western, Kenya; HJF Medical Research International, Kisumu, Western, Kenya; Walter Reed Army Institute of Research - Africa, Kericho, Rift Valley, Kenya; HJF Medical Research International, Tanzania, Mbeya, Mbeya, Tanzania; HJF Medical Research International, Kisumu, Western, Kenya; U.S. Military HIV Research Program, Walter Reed Army Institute of Research AfricaHenry M. Jackson Foundation for the Advancement of Military Medicine, Lagos, Lagos, Nigeria; Henry M. Jackson Foundation for the Advancement of Military Medicine, Bethesda, Maryland; University of California, San Francisco, San Francisco, California; Walter Reed Army Institute of Research, Silver Spring, Maryland; University of Kwazulu-Natal, Durban, KwaZulu-Natal, South Africa

## Abstract

**Background:**

In 2018, dolutegravir (DTG) was recommended by the World Health Organization as the preferred first-line antiretroviral therapy (ART) due to its favourable safety, high resistance barrier, and viral suppression. However, its long-term neurocognitive impact is unclear, especially in low-resource settings. We assessed cognitive performance trajectories before and after DTG transition in adults with HIV.

**Figure:**
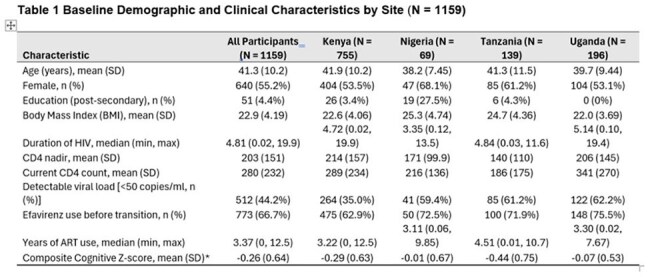

**Figure:**
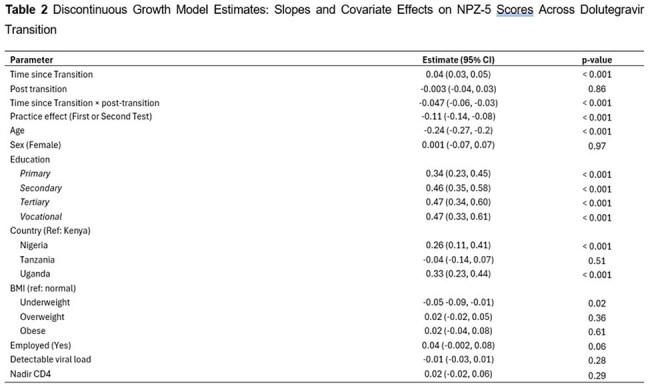

**Methods:**

Data were from AFRICOS, a multicountry cohort in Uganda, Kenya, Nigeria, and Tanzania. Eligible participants had ≥6 annual cognitive assessments (≥ 3 pre- and post-transition). A composite cognitive Z-score averaged z-scores from five tests: WHO Auditory Verbal Learning Test (Trials 1–5, and Delayed Recall), Trails A, and Grooved Pegboard (dominant and non-dominant hands). Reverse-scored tests were adjusted so higher scores reflected better performance. We used a discontinuous growth model to longitudinally estimate cognitive Z-score slopes before and after regimen switch. Models adjusted for age, sex, education, country, BMI, viral load, nadir CD4, and practice effect (i.e., score improvement from repeated testing rather than true change).

**Figure:**
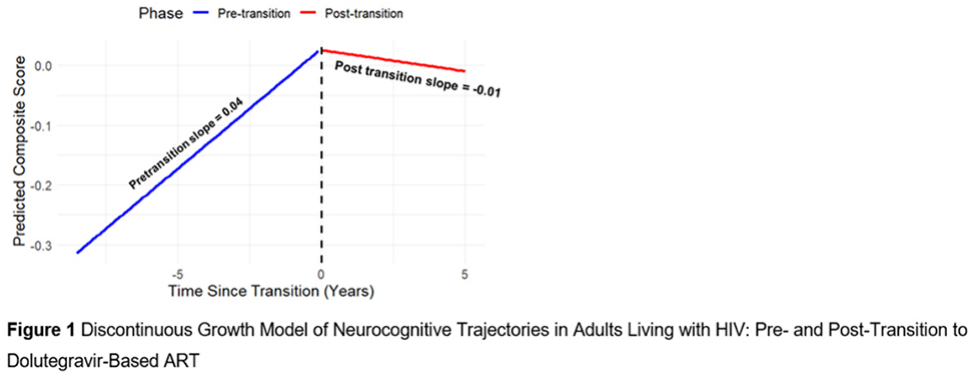

**Figure:**
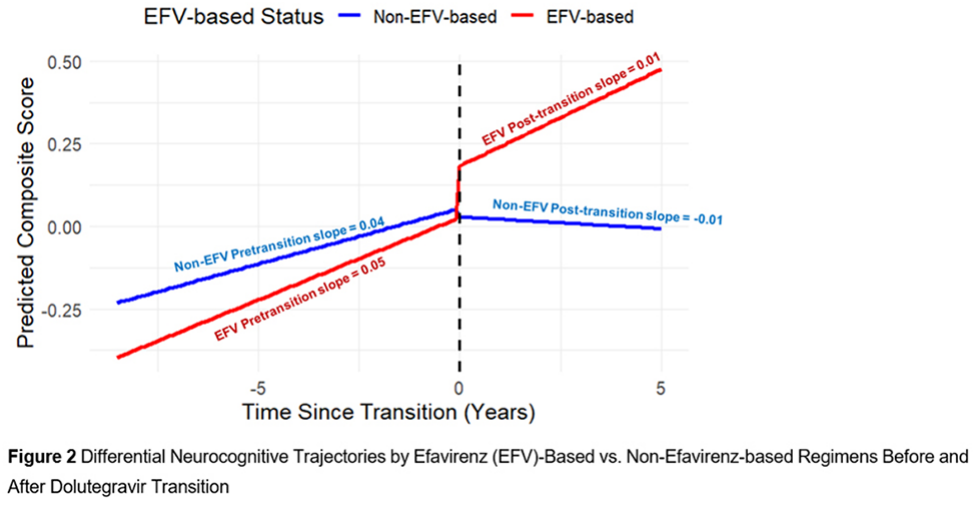

**Results:**

Between 2013 and 2024, 1,159 participants met eligibility criteria for this analysis (mean age 41.3 years; 55.2% female); 66.7% were previously on efavirenz (Table 1). Cognitive scores improved pre-transition (slope = 0.04; 95% CI: 0.03–0.05) but plateaued post-transition (slope ≈ –0.01). The slope change was significant (interaction = –0.047; 95% CI: –0.06, –0.03) (Figure 1). Age, education, BMI, and country were associated with performance (Table 2). Prior efavirenz use was associated with greater gains pre-transition but no significant post-transition advantage (Figure 2).

**Conclusion:**

Cognitive gains slowed post-DTG, especially in older, underweight, and less educated individuals. These findings underscore the need to integrate cognitive monitoring into long-term HIV care. ART transitions may offer a timely opportunity to identify subtle changes and guide supportive care planning.

**Disclosures:**

All Authors: No reported disclosures

